# Supplementary material for: Unlocking Tropical Forest Complexity: How Tree Assemblages in Secondary Forests Boost Biodiversity Conservation
Source: Ecol Evol. 2025 Nov 10;15(11):e72428. doi: 10.1002/ece3.72428 (PMC12600032; doi:10.1002/ece3.72428)
Supplement: Supplementary file 1 — Data S1: ece372428‐sup‐0001‐Supinfo.docx. [file ECE3-15-e72428-s001.docx]

**Supplementary material**

**
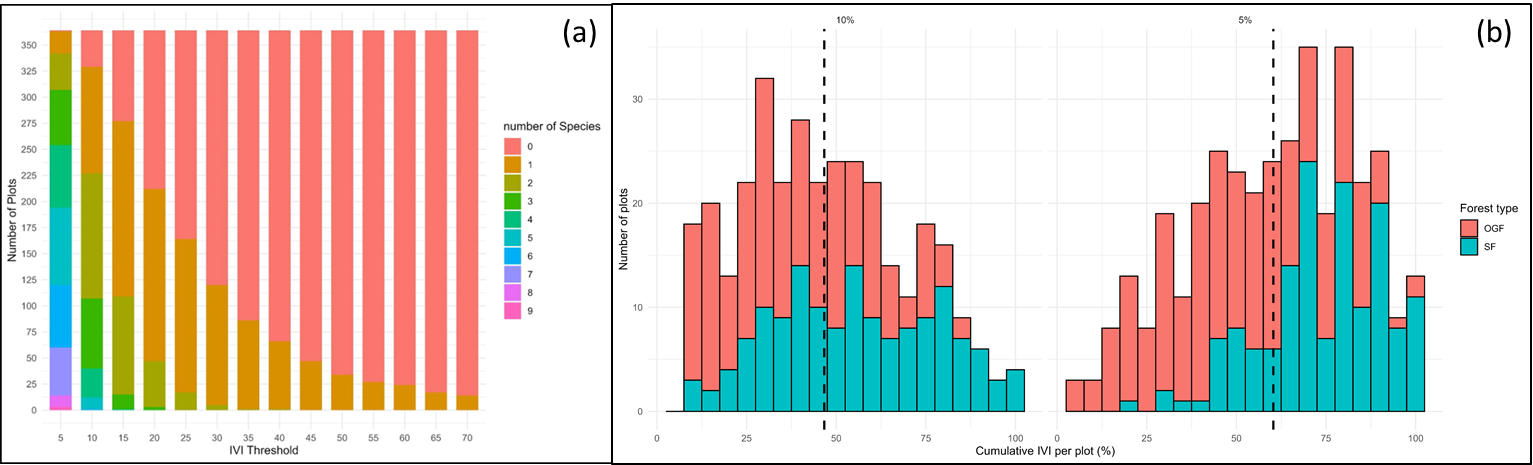
**

**Figure S1.** (a) Fraction of plots with a given number of dominant tree species according to the Importance Value Index (IVI) threshold ; and (b) Distribution of cumulative IVI captured by dominant species according to two thresholds, 5 % for the barplot on the right and 10 % for the one on the left. The y-axis represents cumulative IVI (%), and the x-axis the number of forest plots. Each bar is segmented to show the number of plots by forest type (SF – secondary forest, OGF – old-growth forest). The dashed line indicates the mean cumulative IVI for each threshold.

**
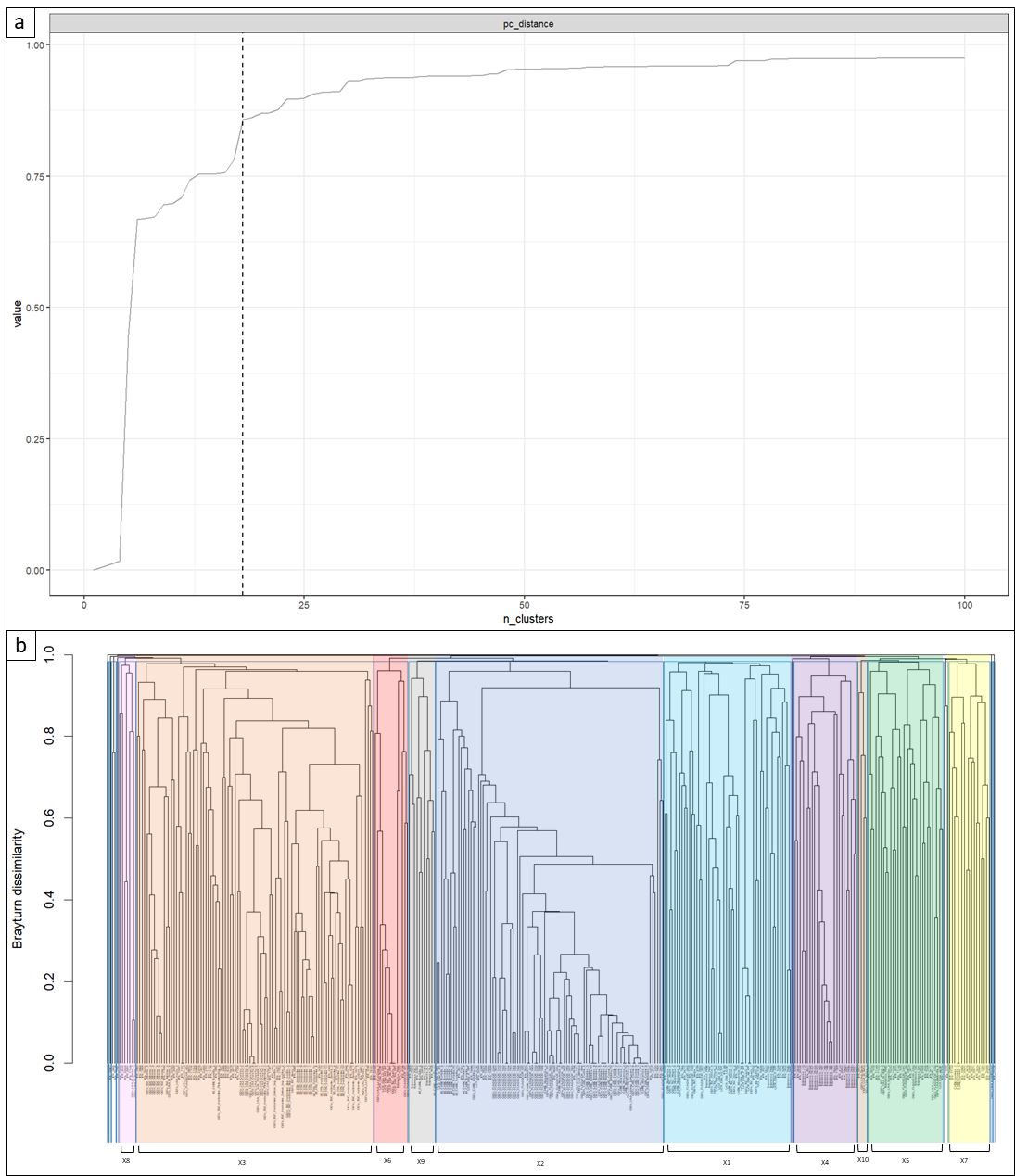
**

**Figure S2.** Results of hierarchical clustering based on the dissimilarity index Bray-Curtis Turnover and UPGMA method. (a) Explained dissimilarity by different number of clusters with the potential optimal number of clusters based on the use of the pc_distance metric in the elbow method, and (b) Hierarchical clustering of 364 sites with the top 10 clusters selected for biogeographical network analysis. The optimal PC_distance is 18, and the cophenetic correlation coefficient of the hierarchical clustering model is 0.48.

**Table S1.** Results of the assessment of isolated sites corresponding to cluster j using Simpson's distance coupled with Bonferroni correction: To distinguish isolated sites with a real floristic specificity from those that simply exhibit a high rate of unique species due to random sampling, a pairwise similarity test was conducted between the isolated sites and the main clusters, using Simpson's distance coupled with a Bonferroni correction. Based on botanical expertise, a similarity threshold of 50% was chosen to allow the association of isolated sites with a similar floristic composition to the main clusters.

| **Cluster j** | **Cluster j'** | **Simpson** |  | **Cluster j** | **Cluster j'** | **Simpson** |  | **Cluster j** | **Cluster j'** | **Simpson** |
| --- | --- | --- | --- | --- | --- | --- | --- | --- | --- | --- |
| C11 | C6 | 0.58 |  | C15 | C3 | 0.78 |  | C18 | C3 | 1.00 |
|  | C1 | 0.46 |  |  | C8 | 0.48 |  |  | C1 | 0.33 |
|  | C2 | 0.29 |  |  | C6 | 0.26 |  |  | C6 | 0.33 |
|  | C5 | 0.21 |  |  | C1 | 0.17 |  |  |  |  |
|  | C9 | 0.17 |  |  | C2 | 0.09 |  |  |  |  |
|  | C3 | 0.17 |  |  | C5 | 0.04 |  |  |  |  |
|  | C8 | 0.17 |  | C16 | C6 | 0.41 |  |  |  |  |
|  | C7 | 0.13 |  |  | C9 | 0.29 |  |  |  |  |
| C12 | C3 | 0.25 |  |  | C3 | 0.29 |  |  |  |  |
|  | C7 | 0.25 |  |  | C1 | 0.29 |  |  |  |  |
|  | C8 | 0.25 |  |  | C2 | 0.24 |  |  |  |  |
| C13 | C1 | 0.38 |  |  | C5 | 0.18 |  |  |  |  |
|  | C5 | 0.33 |  |  | C7 | 0.06 |  |  |  |  |
|  | C8 | 0.33 |  | C17 | C5 | 0.65 |  |  |  |  |
|  | C6 | 0.30 |  |  | C10 | 0.51 |  |  |  |  |
|  | C4 | 0.20 |  |  | C8 | 0.21 |  |  |  |  |
|  | C9 | 0.18 |  |  | C4 | 0.21 |  |  |  |  |
|  | C7 | 0.18 |  |  | C9 | 0.16 |  |  |  |  |
|  | C2 | 0.15 |  |  | C6 | 0.15 |  |  |  |  |
|  | C3 | 0.05 |  |  | C1 | 0.14 |  |  |  |  |
| C14 | C3 | 0.56 |  |  | C2 | 0.13 |  |  |  |  |
|  | C2 | 0.11 |  |  | C7 | 0.13 |  |  |  |  |
|  | C6 | 0.11 |  |  | C3 | 0.02 |  |  |  |  |

**Table S2.** List of contributive species ($\rho_{ij}$ ≥ 1.96) by in situ cluster.


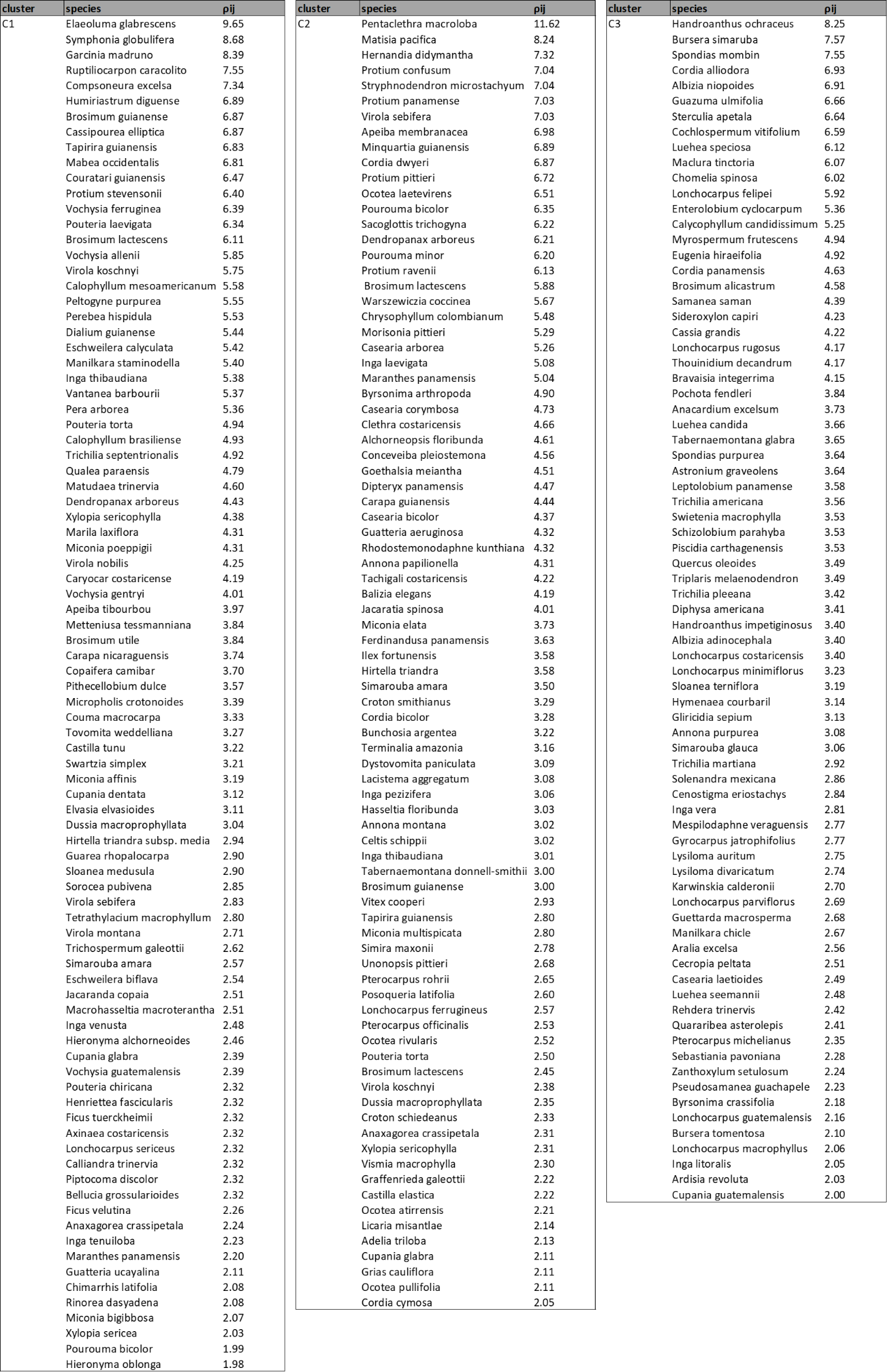


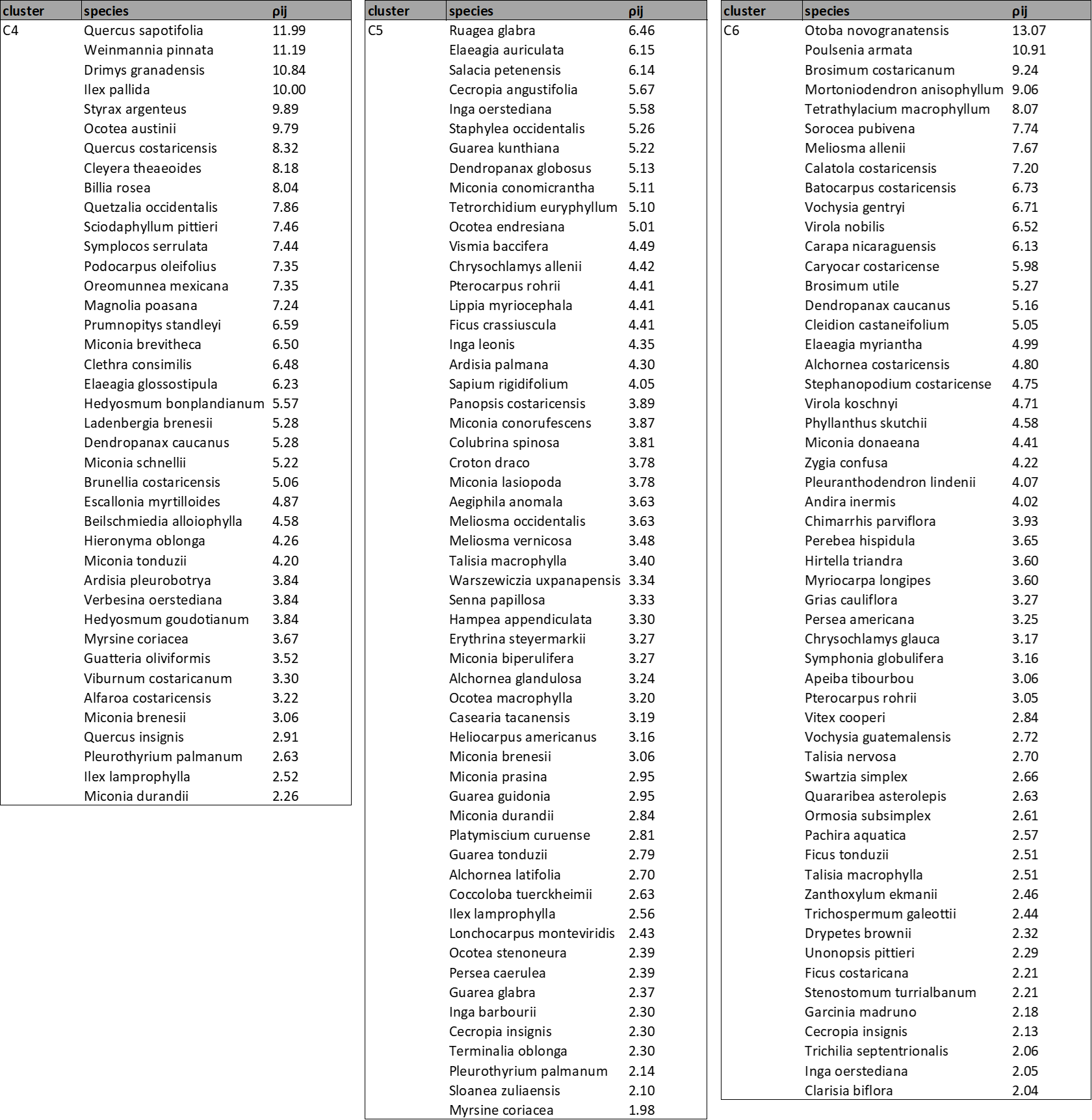


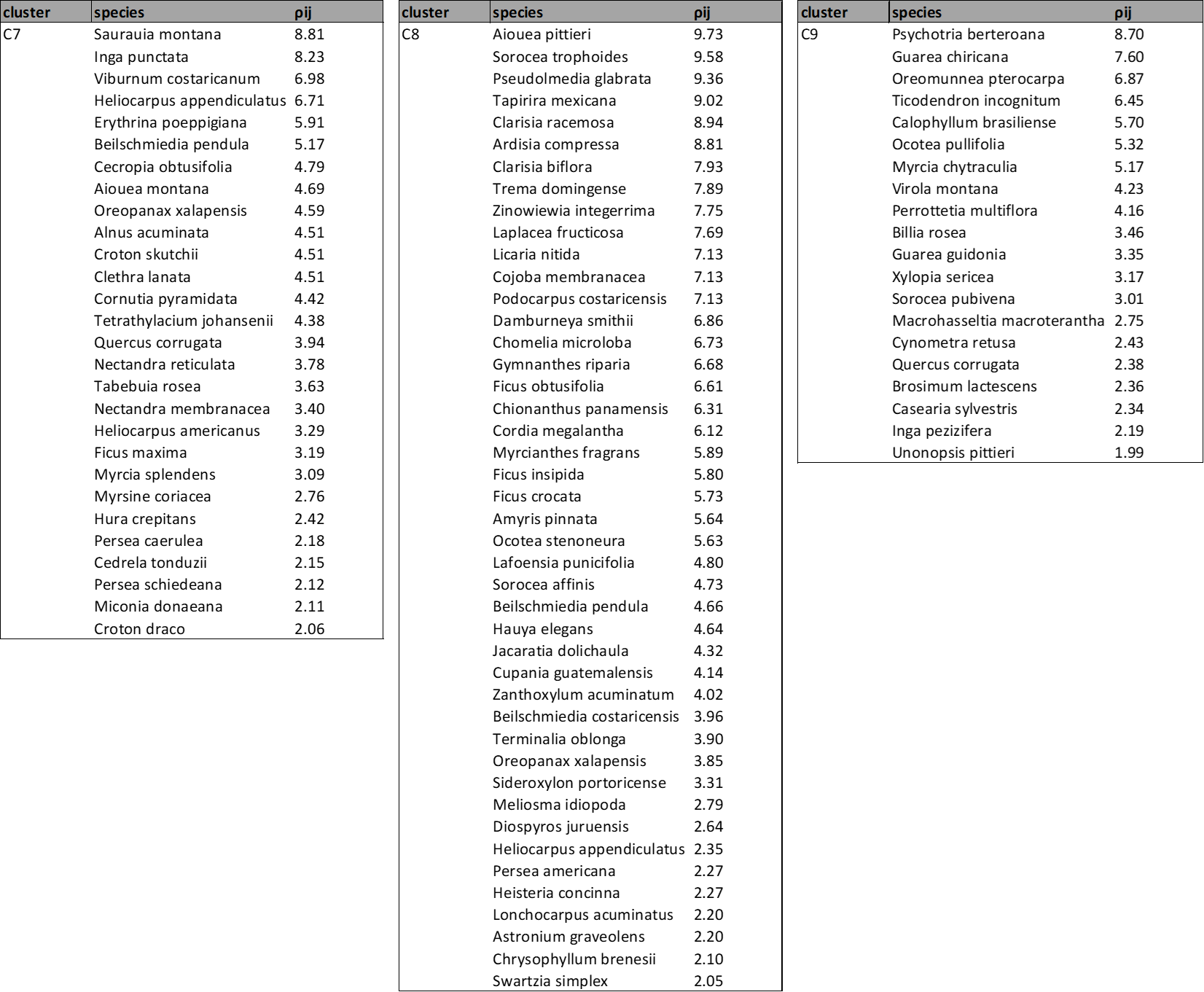


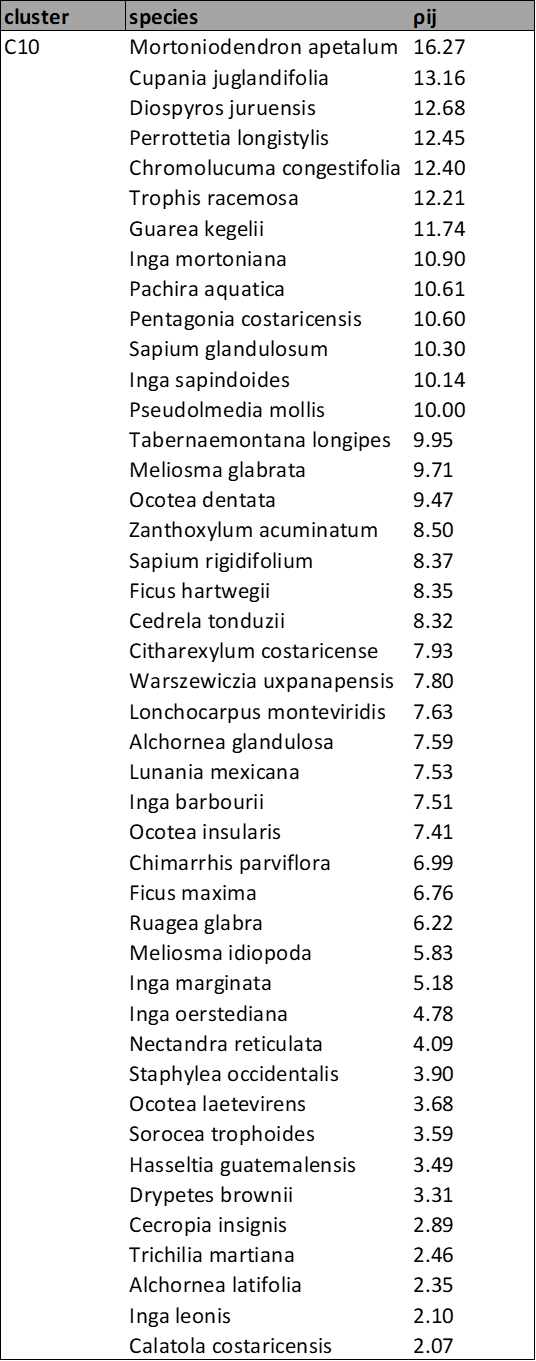


**Table S3.** The matrix of mean fractions of contribution to the cluster j from contributive species ($\rho_{ij} \geq1.96$) that also contribute significantly to cluster $j'$ ($\lambda_{jj'}$). The grey values in the matrix represent $\lambda_{jj}$, the specificity of cluster $j$. These fractions are expressed as percentages with a vector $\lambda_{j}$ for a given cluster that sums to 1.

| **Matrix λ jj'** | | | | | | | | | | |
| --- | --- | --- | --- | --- | --- | --- | --- | --- | --- | --- |
|  | **C2** | **C9** | **C3** | **C7** | **C1** | **C5** | **C6** | **C8** | **C4** | **C10** |
| **C2** | 0.88 | 0.02 | 0.00 | 0.00 | 0.07 | 0.01 | 0.02 | 0.00 | 0.00 | 0.01 |
| **C9** | 0.08 | 0.69 | 0.00 | 0.03 | 0.09 | 0.01 | 0.05 | 0.00 | 0.05 | 0.00 |
| **C3** | 0.00 | 0.00 | 0.99 | 0.00 | 0.00 | 0.00 | 0.00 | 0.00 | 0.00 | 0.00 |
| **C7** | 0.00 | 0.03 | 0.00 | 0.78 | 0.00 | 0.03 | 0.02 | 0.07 | 0.02 | 0.05 |
| **C1** | 0.04 | 0.02 | 0.00 | 0.00 | 0.88 | 0.00 | 0.06 | 0.00 | 0.00 | 0.01 |
| **C5** | 0.00 | 0.01 | 0.00 | 0.01 | 0.00 | 0.85 | 0.02 | 0.03 | 0.03 | 0.06 |
| **C6** | 0.02 | 0.01 | 0.00 | 0.00 | 0.10 | 0.02 | 0.80 | 0.01 | 0.00 | 0.03 |
| **C8** | 0.00 | 0.00 | 0.01 | 0.04 | 0.01 | 0.03 | 0.02 | 0.86 | 0.00 | 0.04 |
| **C4** | 0.00 | 0.02 | 0.00 | 0.01 | 0.00 | 0.06 | 0.01 | 0.00 | 0.90 | 0.01 |
| **C10** | 0.01 | 0.00 | 0.01 | 0.01 | 0.02 | 0.08 | 0.03 | 0.02 | 0.00 | 0.82 |

**Table S4.** List of contributive species that contribute to more than one in situ cluster, i.e. with a relative contribution $\hat{\rho}_{ij}^{+}$ of a species $i$ to a cluster $j$ less than 1 : 96 species contribute to more than one cluster, with 7 contributing to three clusters and 89 to two clusters, based on $\rho_{ij} \geq1.96$.

| **Species** | **Clusters** | | |
| --- | --- | --- | --- |
| *Cecropia insignis* | C5 | C6 | C10 |
| *Brosimum lactescens* | C2 | C9 | C1 |
| *Sorocea pubivena* | C9 | C1 | C6 |
| *Pterocarpus rohrii* | C2 | C5 | C6 |
| *Virola koschnyi* | C2 | C1 | C6 |
| *Unonopsis pittieri* | C2 | C9 | C6 |
| *Swartzia simplex* | C1 | C6 | C8 |
| *Virola sebifera* | C2 | C1 |  |
| *Simarouba amara* | C2 | C1 |  |
| *Guarea guidonia* | C9 | C5 |  |
| *Vochysia guatemalensis* | C1 | C6 |  |
| *Dendropanax arboreus* | C2 | C1 |  |
| *Ficus maxima* | C7 | C10 |  |
| *Ocotea pullifolia* | C2 | C9 |  |
| *Tapirira guianensis* | C2 | C1 |  |
| *Staphylea occidentalis* | C5 | C10 |  |
| *Inga pezizifera* | C2 | C9 |  |
| *Calophyllum brasiliense* | C9 | C1 |  |
| *Inga oerstediana* | C5 | C10 |  |
| *Nectandra reticulata* | C7 | C10 |  |
| *Astronium graveolens* | C3 | C8 |  |
| *Apeiba tibourbou* | C1 | C6 |  |
| *Heliocarpus appendiculatus* | C7 | C8 |  |
| *Dussia macroprophyllata* | C2 | C1 |  |
| *Drypetes brownii* | C6 | C10 |  |
| *Pouteria torta* | C2 | C1 |  |
| *Xylopia sericophylla* | C2 | C1 |  |
| *Cupania glabra* | C2 | C1 |  |
| *Brosimum guianense* | C2 | C1 |  |
| *Inga thibaudiana* | C2 | C1 |  |
| *Alchornea latifolia* | C5 | C10 |  |
| *Beilschmiedia costaricensis* | C5 | C8 |  |
| *Maranthes panamensis* | C2 | C1 |  |
| *Pachira aquatica* | C6 | C10 |  |
| *Ocotea laetevirens* | C2 | C10 |  |
| *Myrcia chytraculia* | C9 | C5 |  |
| *Persea americana* | C6 | C8 |  |
| *Hirtella triandra* | C2 | C6 |  |
| *Garcinia madruno* | C1 | C6 |  |
| *Brosimum utile* | C1 | C6 |  |
| *Xylopia sericea* | C9 | C1 |  |
| *Symphonia globulifera* | C1 | C6 |  |
| *Caryocar costaricense* | C1 | C6 |  |
| *Miconia brenesii* | C5 | C4 |  |
| *Calatola costaricensis* | C6 | C10 |  |
| *Guatteria oliviformis* | C5 | C4 |  |
| *Trichilia martiana* | C3 | C10 |  |
| *Zanthoxylum acuminatum* | C8 | C10 |  |
| *Inga barbourii* | C5 | C10 |  |
| *Warszewiczia uxpanapensis* | C5 | C10 |  |
| *Myrsine coriacea* | C7 | C4 |  |
| *Cordia cymosa* | C2 | C5 |  |
| *Grias cauliflora* | C2 | C6 |  |
| *Anaxagorea crassipetala* | C2 | C1 |  |
| *Chimarrhis parviflora* | C6 | C10 |  |
| *Billia rosea* | C9 | C4 |  |
| *Lonchocarpus monteviridis* | C5 | C10 |  |
| *Quararibea asterolepis* | C3 | C6 |  |
| *Meliosma idiopoda* | C8 | C10 |  |
| *Hasseltia guatemalensis* | C5 | C10 |  |
| *Beilschmiedia pendula* | C7 | C8 |  |
| *Oreopanax xalapensis* | C7 | C8 |  |
| *Chrysophyllum brenesii* | C1 | C8 |  |
| *Sorocea trophoides* | C8 | C10 |  |
| *Ruagea glabra* | C5 | C10 |  |
| *Sapium rigidifolium* | C5 | C10 |  |
| *Heliocarpus americanus* | C7 | C5 |  |
| *Pleurothyrium palmanum* | C5 | C4 |  |
| *Terminalia oblonga* | C5 | C8 |  |
| *Damburneya cufodontisii* | C9 | C4 |  |
| *Miconia durandii* | C5 | C4 |  |
| *Vitex cooperi* | C2 | C6 |  |
| *Vochysia gentryi* | C1 | C6 |  |
| *Cedrela tonduzii* | C7 | C10 |  |
| *Miconia donaeana* | C7 | C6 |  |
| *Persea schiedeana* | C9 | C7 |  |
| *Perrottetia multiflora* | C9 | C6 |  |
| *Quercus corrugata* | C9 | C7 |  |
| *Viburnum costaricanum* | C7 | C4 |  |
| *Carapa nicaraguensis* | C1 | C6 |  |
| *Tetrathylacium macrophyllum* | C1 | C6 |  |
| *Virola montana* | C9 | C1 |  |
| *Diospyros juruensis* | C8 | C10 |  |
| *Ocotea stenoneura* | C5 | C8 |  |
| *Sideroxylon portoricense* | C5 | C8 |  |
| *Alchornea glandulosa* | C5 | C10 |  |
| *Perebea hispidula* | C1 | C6 |  |
| *Trichospermum galeottii* | C1 | C6 |  |
| *Dendropanax caucanus* | C6 | C4 |  |
| *Macrohasseltia macroterantha* | C9 | C1 |  |
| *Croton draco* | C7 | C5 |  |
| *Virola nobilis* | C1 | C6 |  |
| *Persea caerulea* | C7 | C5 |  |
| *Talisia macrophylla* | C5 | C6 |  |
| *Inga leonis* | C5 | C10 |  |
| *Ilex lamprophylla* | C5 | C4 |  |

**Table S5.** Mean values and standard deviation (SD) of the environmental variables used to characterise the 7 modelled forest ecosystems. q25, q50 and q75 represent the 25th, 50th, and 75th percentiles of the variables measured across the forest segments, respectively. Topographic variables: DEM, representing elevation in meters, and slope, expressed in degrees. Climatic variables: PRSea, the coefficient of variation of precipitation seasonality, and anPR, the mean annual precipitation, expressed in mm/year. Edaphic variables: pH and Cation Exchange Capacity (CEC) expressed in mmolc/kg, at a depth of 30 cm. Vegetation dynamics variable: NDWIw, Normalised Difference Water Index of wet season. Importance values are derived from the permutation of variables in the model.

| **Clusters** | **q50.DEM** | | **Q75.anPR** | | **Q25.PRSea** | | | | **q25.NDWIw** | | | **q50.pH30** | | | **q25.Slope** | | | **q50.CEC30** | |
| --- | --- | --- | --- | --- | --- | --- | --- | --- | --- | --- | --- | --- | --- | --- | --- | --- | --- | --- | --- |
|  | **mean** | **sd** | **mean** | **sd** | | **mean** | **sd** | **mean** | | **sd** | **mean** | | **sd** | **mean** | | **sd** | **mean** | | **sd** |
| C1 | 504 | 361 | 3611 | 707 | | 51 | 13 | 0.31 | | 0.11 | 5.21 | | 0.62 | 10 | | 8 | 134 | | 44 |
| C2 | 141 | 205 | 3522 | 726 | | 34 | 9 | 0.31 | | 0.07 | 5.27 | | 0.62 | 4 | | 4 | 132 | | 49 |
| C3 | 258 | 261 | 2308 | 542 | | 75 | 14 | 0.22 | | 0.09 | 5.73 | | 0.69 | 8 | | 7 | 212 | | 60 |
| C4 | 2269 | 463 | 3804 | 614 | | 45 | 10 | 0.37 | | 0.08 | 5.12 | | 0.17 | 20 | | 8 | 192 | | 39 |
| C5 | 1142 | 509 | 3616 | 547 | | 48 | 13 | 0.37 | | 0.05 | 5.34 | | 0.2 | 14 | | 7 | 171 | | 45 |
| C6 | 296 | 206 | 3666 | 667 | | 65 | 8 | 0.39 | | 0.03 | 5.28 | | 0.29 | 13 | | 8 | 183 | | 42 |
| C7 | 1360 | 372 | 3245 | 549 | | 55 | 12 | 0.26 | | 0.08 | 5.29 | | 0.31 | 16 | | 8 | 162 | | 33 |


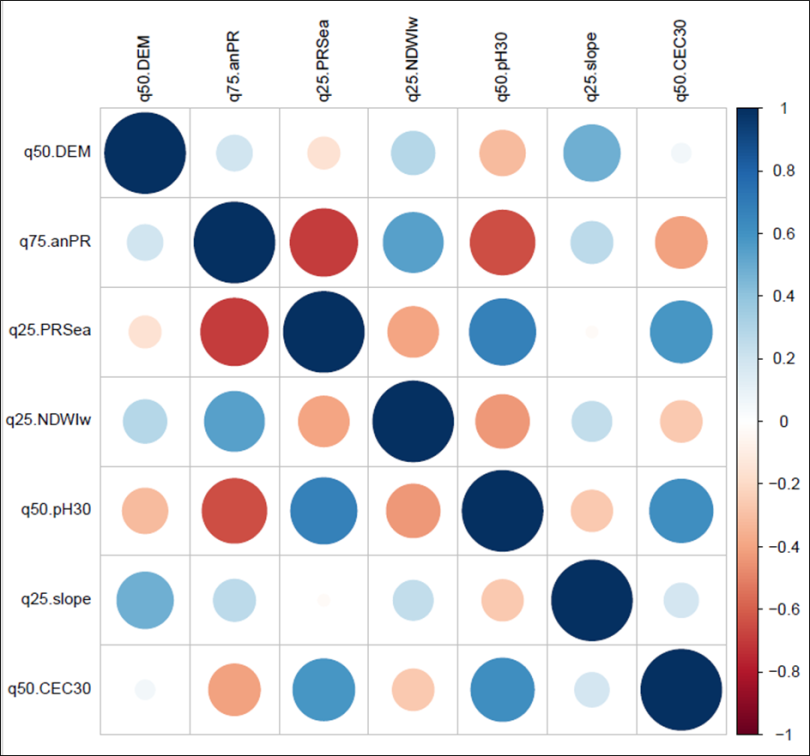


**Figure S3**. Correlation matrix of key variables selected for the Random Forest model from Pearson correlation: Topographic variables. q25, q50 and q75 represent the 25th, 50th, and 75th percentiles of the variables measured across the forest segments, respectively. DEM, representing elevation in meters, and Slope, expressed in degrees. Climatic variables: PRSea, the coefficient of variation of precipitation seasonality, and anPR, the mean annual precipitation, expressed in mm/year. Edaphic variables: pH and CEC, Cation Exchange Capacity expressed in mmolc/kg, at a depth of 30 cm. Vegetation dynamics variable : NDWIw, Normalized Difference Water Index of wet season.


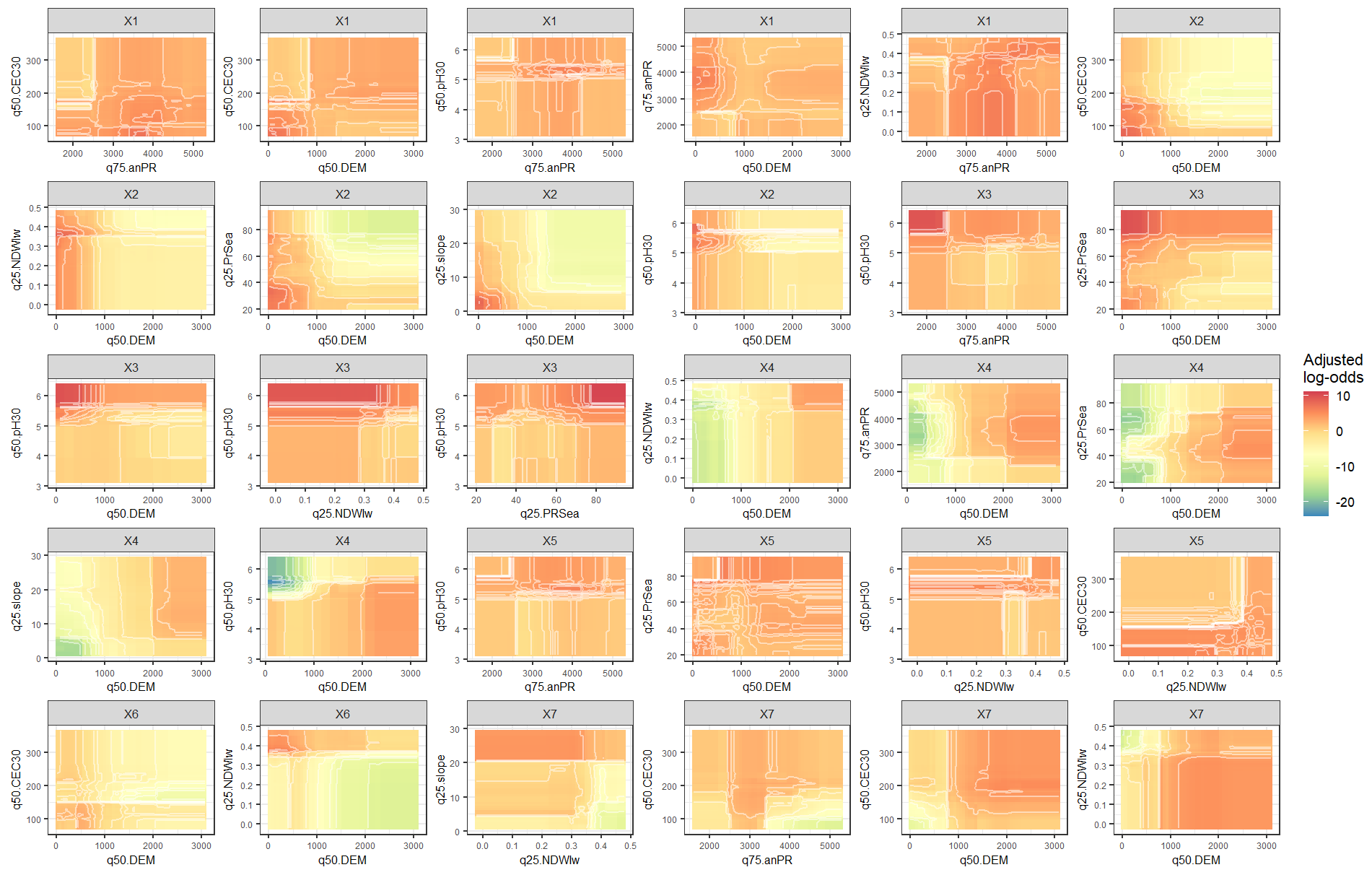


**Figure S4.** Partial dependence plots between two variables to analyse the main marginal effects of the most important predictive variables for each cluster in adjusted log-odds. Positive adjusted log-odds indicate a high probability of classification into the given cluster, zero represents a 50% probability, and negative values indicate a low or near zero probability of classification.

**Table S6.** Distribution of plots by number and proportion (%) along the altitudinal gradient in Costa Rica.

| **Plots** | |  | **Altitudinal range** |
| --- | --- | --- | --- |
| **Number** | **Proportion** |  |  |
| 250 | 69.64 |  | [0, 500) |
| 51 | 14.21 |  | [500, 1000) |
| 14 | 3.90 |  | [1000, 1500) |
| 42 | 11.70 |  | [1500, 2000) |
| 2 | 0.56 |  | [2000, 2500) |

**Table S7**. Correspondence table between local and national forest characterisations of the lowland forests of Osa Peninsula. The local characterisation, conducted by Hofhansl et al. (2019) from some sites included in our analysis, highlights the key role of tree species that contribute to the national forest ecosystems present in this region. In these sites, ecosystem WSE dominates, although ecosystems LWE-C and TWPE-P are also represented. They identify four forest-types (Ridge, Salope, Ravine and Secondary) sharing many dominant species based on the 10 most dominant species of each type. This table illustrates the complexity of delineating forest-types in the peninsula, reflected by the overlap of contributive species shared by different forest ecosystems identified at the national level.

| **Dominant Species** | **Local characterisation** | **National Characterisation** |
| --- | --- | --- |
| *Vochysia ferruginea* | Ridge forests | WSE |
| *Pourouma bicolor* | Ridge forests | WSE, LWE-C |
| *Compsoneura excelsa* | Ridge and Slope forests | WSE, LWE-C, TWPE-P |
| *Mabea occidentalis* | Ridge and, Slope forests | WSE, LWE-C, TWPE-P |
| *Tapirira guianensis* | Ridge and Slope forests | WSE, LWE-C, TWPE-P |
| *Otoba novogranatensis* | Slope and Ravine forests | TWPE-P |
| *Sorocea pubivena* | Slope and Ravine forests | WSE, TWPE-P |
| *Goethalsia meiantha* | Ravine and Secondary forests | LWE-C |
| *Apeiba tibourbou* | Secondary forests | WSE, TWPE-P |
| *Hieronyma alchorneoides* | Secondary forests | WSE, TWPE-P |
| *Castilla tunu* | Secondary forests | WSE, TWPE-P |
| *Alchornea costaricensis* | Secondary forests | WSE, TWPE-P |
| *Tetrathylacium macrophyllum* | Slope, Ravine and Secondary forests | WSE, TWPE-P |
| *Symphonia globulifera* | Ridge, Slope and Ravine forests | WSE, TWPE-P |
| *Carapa nicaraguensis* | Ridge, Slope, Ravine and secondary forests | WSE, LWE-C, TWPE-P |

**Table S8**. Description based on botanical expertise of the seven main modelled national forest ecosystems.

| **Ecosystems** | **Description** |
| --- | --- |
| Wet Seasonal Evergreen forest (WSE) | This forest ecosystem is characterised by a high composition of tree species, typical of old-growth forests in a well-preserved state. Its indicator species commonly occupy the canopy or emerge above it and are significantly abundant within the ecosystem. While no particular species dominates, species diversity and abundance are heterogeneous. This species composition and/or mix is consistent with the altitudinal range in which they are distributed, mainly between 0 and 700 m, and they predominantly occupy very humid seasonal lowland forests. Biogeographically, this ecosystem has two cores of tree diversity, the most diverse located in the Osa Peninsula and another, more isolated, restricted to the northernmost region of Costa Rica. Both cores (northern zone/Osa Peninsula) share a significant number of tree species, yet also contain unique, non-overlapping species specific to each biogeographical region. These ecosystems typically occupy areas with rather irregular topography. |
| Lowland Wet Evergreen forest of Caribbean slope (LWE-C) | This forest ecosystem is characterised by a tree species composition that is clearly dominated by the abundance or presence of Pentaclethra macroloba. Forests with a high presence of Pentaclethra macroloba exhibit a very distinctive associated tree diversity, both in the canopy and the understory, with a strong dominance of arboreal or shrubby palms. Although this ecosystem has high species diversity, other tree species (besides Pentaclethra macroloba) can also become dominant in the overall forest structure. The species composition of this ecosystem results from forests subject to intervention or logging, as evidenced by the presence of a number of fast-growing species interspersed, which are more typical of disturbed forests or late-secondary to old secondary forests. This ecosystem prefers areas with relatively flat or undulating topography, as well as alluvial plains. |
| Lowland Dry-to-Moist Deciduous-to-Semi-deciduous forest (LDM-DS) | This forest ecosystem is characterised by a composition of tree species that are typical of, or commonly found in, deciduous or semi-deciduous dry forests, interspersed with some evergreen species scattered throughout the landscape or associated with riparian forests. Its diversity is heterogeneous but not particularly high, as it exhibits strong dominance by species from the Fabaceae family (primarily), as well as Bignoniaceae, Malvaceae, Burseraceae, Anacardiaceae, among others. This ecosystem experiences the highest levels of climatic seasonality in the country, with the greatest number of dry months. As a result, this climatic factor is the primary driver of the dominant floristic pattern. Additionally, this ecosystem has undergone a long history of anthropogenic use and impacts (particularly fire), which have significantly altered its original species composition. In general, this ecosystem hosts a tree species composition with a broad geographical distribution along the Pacific coast of Mesoamerica. In Costa Rica, in particular, it is closely associated with the climatic seasonality gradient along the Pacific coast. Notably, some species from this ecosystem also occur in other, distant and ecologically distinct ecosystems (secondary forests) within the country, largely as a consequence of the historical movement of cattle ranching across the landscape. |
| Mountain Oak Rainforest (MOR) | This forest ecosystem is characterised by a tree species composition largely dominated by oak species (Quercus spp.), forming the well-known associations called "oak forests", where, depending on the altitudinal gradient, one, two, or more Quercus species dominate the forest structure. The sampling plots were mainly concentrated within the 1500–2000 m elevation band, primarily on the Pacific slope, where the forests are seasonal evergreen. Consequently, the recorded composition is representative of this altitudinal range. Additionally, most of the sampled plots correspond to old-growth forests. The largest expanse of oak forests typically occurs above 2000 m. |
| Premontane-to-mountain Mixed-to-evergreen Cloud forest of Caribbean slope (PMC-C) | This forest ecosystem is characterised by a composition of tree species from mid-elevations (1410–1957 m, based on the sampling) on the Caribbean slope. The inclusion of several plots below 500 m, all classified as Secondary Forest, affects the definition and characterisation of this ecosystem’s tree composition. Moreover, most of these Secondary Forest plots actually belong to the Caribbean lowland ecosystem. |
| Transitional wet premontane evergreen forest of the Pacific slope (TWPE-P) | *Otoba novogranatensis* and *Poulsenia armata*, indicators of forest maturity, are the most contributive species. However, this ecosystem is found in forests that have been disturbed by selective logging, which explains the presence of many species typical of secondary or disturbed forests among the contributive species. This ecosystem, primarily located in the Osa Peninsula, represents an extension of the LWE-C ecosystem, with many common contributive species. It is likely that this discrimination is due to the inclusion in the analysis of 11 LWE-C plots located above 700 m in altitude, 8 of which come from secondary forests. The tree composition model above this altitude differs significantly from the LWE-C type. |
| Premontane-to-mountain Mixed-to-evergreen Cloud forest of Pacific slope (PMC-P) | This forest ecosystem is characterised by a tree species composition dominated by secondary forest species, as the majority or nearly all of the plots belong to SF. Additionally, most plots are located in the mid-elevation band (1111 m) on the Pacific slope, where the vegetation cover is highly fragmented due to coffee cultivation. This elevation band also experiences a climate with a strongly marked seasonality. |
